# Long-Term Calorie Restriction Alters Anxiety-like Behaviour and the Brain and Adrenal Gland Transcriptomes of the Ageing Male Rat

**DOI:** 10.3390/nu14214670

**Published:** 2022-11-04

**Authors:** Antonina Govic, Helen Nasser, Elizabeth A. Levay, Matt Zelko, Esmaeil Ebrahimie, Manijeh Mohammadi Dehcheshmeh, Stephen Kent, Jim Penman, Agnes Hazi

**Affiliations:** 1 School of Psychology and Public Health, La Trobe University, Melbourne, VIC 3010, Australia; 2 Epigenes Australia Pty Ltd., Melbourne, VIC 3010, Australia; 3 Genomics Research Platform, School of Agriculture, Biomedicine and Environment, La Trobe University, Melbourne, VIC 3000, Australia; 4 School of Animal and Veterinary Sciences, The University of Adelaide, Adelaide, SA 5371, Australia; 5 School of BioSciences, The University of Melbourne, Melbourne, VIC 3010, Australia

**Keywords:** calorie restriction, food restriction, anxiety-like behaviour, ageing, gene expression, transcriptomics, hypothalamus, amygdala, pituitary, adrenal glands

## Abstract

Further examination of the molecular regulators of long-term calorie restriction (CR), reported to have an anxiolytic effect, may highlight novel therapeutic targets for anxiety disorders. Here, adult male Hooded Wistar rats were exposed to a 25% CR whilst anxiety-like behaviour was assessed at 6-, 12-, and 18-months of age via the elevated plus maze, open field, and acoustic startle tests. Next-generation sequencing was then used to measure transcriptome-wide gene expression in the hypothalamus, amygdala, pituitary, and adrenal glands. Results showed an anxiolytic behavioural profile across early, middle, and late adulthood by CR, with the strongest effects noted at 6-months. Transcriptomic analysis by seven attribute weighting algorithms, including Info Gain Ratio, Rule, Chi Squared, Gini Index, Uncertainty, Relief, and Info Gain, led to the development of a signature of long-term CR, independent of region. Complement C1q A chain (*C1qa*), an extracellular protein, expression was significantly decreased by CR in most regions examined. Furthermore, text mining highlighted the positive involvement of *C1qa* in anxiety, depression, neurodegeneration, stress, and ageing, collectively identifying a suitable biomarker candidate for CR. Overall, the current study identified anxiety-related phenotypic changes and a novel transcriptome signature of long-term CR, indicating potential therapeutic targets for anxiety, depression, and neurodegeneration.

## 1. Introduction

Ageing is accompanied by physical and cognitive deterioration and an increased risk of disease. Epidemiological and clinical findings also indicate that psychiatric disorders, such as depression and anxiety, are highly prevalent in the elderly with rates of anxiety as high as 15% in community samples and 28% in clinical samples [1,2]. Commensurate with a bourgeoning elderly population worldwide, prevalence rates of anxiety disorders are increasing, along with significant individual and societal costs [3]. Therefore, much research and clinical effort have been directed towards interventions for anxiety disorders in the elderly, though treatment response rates are lower in this population relative to younger adults [4].

Increasing evidence points to the deterministic role of diet, specifically, the overconsumption of energy-dense foods, in the pathogenesis of chronic diseases, as well as accelerated ageing [5]. Conversely, calorie restriction (CR), defined as a reduction in *ad libitum* intake without malnutrition, has consistently been demonstrated to reduce the incidence of age-related diseases and retard senescence, thereby extending health- and lifespan in multiple animal species [6,7,8]. As well as these physiological benefits, CR attenuates age-related cognitive decline in both healthy and clinical human populations (see [9] for a narrative review). Whether CR exerts similar improvements in emotional functioning in an ageing population is not well known.

Research in rodents demonstrates that short-term CR initiated in adulthood consistently reduces anxiety-like behaviour in tests of forced exploration [10,11,12,13,14,15] and in non-locomotive-based tests of anxiety, such as the acoustic startle test [16,17]. However, results from the few longer-term (>1-month) CR studies conducted report contradictory findings with respect to the behavioural phenotype expressed throughout the lifetime. Specifically, CR has been reported to reduce anxiety-like behaviours in female mice during early adulthood but not late in life [18], or conversely during late adulthood but not young adulthood in male mice [19]. Moreover, a recent study found that a late-onset CR (15, 18, 21, or 24 months) of 3- or 6-months duration results in divergent behavioural profiles in ageing male rats [20]. Methodological differences in sex, species, CR onset, duration, and degree likely account for these conflicting findings, further underscoring the importance in systematically determining the effect of CR on anxiety-like behaviour across the adult lifespan of animals.

The mechanisms mediating the anxiolytic-like effects of CR have not been fully elucidated. It is evident; however, that CR leads to altered gene expression in several brain regions and neuromodulators implicated in the regulation of stress and anxiety states. For instance, short-term CR results in a striking degree of altered gene expression in the amygdala of mice who demonstrate an anxiolytic behavioural profile [15]. Gene expression changes in the hypothalamus, another key brain region involved in the regulation of anxiety [21], are also evident in mice [22] and rats [23] undergoing a short-term mild to moderate (5% to 30%) calorie reduced diet. Moreover, many of the identified genes altered as a consequence of CR are associated with the Hypothalamic-Pituitary-Adrenal (HPA) axis [15,22,24], also heavily implicated in the pathogenesis of anxiety disorders (see [25] for a review). Whether these gene expression changes are a transient effect of CR remains to be determined. Furthermore, to our knowledge, no studies have concurrently examined the effect of long-term CR and anxiety-like behaviour and gene expression profiles in other brain areas or tissue associated with the regulation of anxiety in ageing rats.

To this end, this study aimed to characterise the effect of an adult-onset long-term chronic CR on anxiety-associated behaviour in the ageing male rat. Additionally, with the further aim of identifying transcriptomic signatures and signalling pathways of long-term CR and identifying possible novel candidate genes, we examined the transcriptome-wide gene expression patterns of the hypothalamus, amygdala, pituitary, and adrenal glands, key areas involved in the regulation of anxiety. Then, we employed literature mining to link the responding genes to long-term CR with anxiety, behaviour, and ageing.

## 2. Materials and Methods

### 2.1. Animals

Adult (12- to 13-week-old; 300 ± 7.3 g at onset of experiment) male specific pathogen-free Hooded Wistar rats (Animal Resources Centre, Western Australia) were group housed (3–4 rats/cage) in large polypropylene basin cages (56.5 × 38.5 × 19.5 cm, l × w × h). All rats were maintained on a reverse 12:12 h light: dark cycle (lights off at 1100 h), at an ambient temperature (24 ± 1 °C), and provided with a hide-box, wood shaving and shredded paper as bedding. Rats were provided with standard rat chow (Barastoc, Ridley Corporation, Victoria, Australia) and were allowed free access to tap water throughout experimentation. All procedures were conducted in accordance with the National Health and Medical Research Council of Australia Code of Practice for the Care of Experimental Animals and approval gained by RMIT University Animal Ethics Committee (approval number 1402).

### 2.2. Dietary Treatment

Rats were randomly assigned (body weight balanced) to two groups (*n* = 7–8/group): control and calorie restriction (CR). Controls were allowed *ad libitum* access to food throughout experimentation. CR rats received 75% (25% restriction) of the amount of food consumed by the age- and weight-matched control rats delivered daily within the hour preceding lights out (1000–1100 h). The food intake of the CR group was initially determined by calculating the food intake of all rats across the last 48-h period of acclimation, and thereafter from the control group over a 48-h period on a monthly basis. Mean daily control food intake at 6 and 12 months of age was stable at 24.7 to 23.4 g/day/rat, while intake decreased at 18 months to 19.9 g/rat/day. Consequently, mean CR intake ranged from ~18.5 to 15.3 g/rat/day throughout the experimental period. The dietary regimen employed involved an overall reduction in food intake and the composition of the diet has been previously reported [14].

### 2.3. Behavioural Testing

Behavioural testing in the elevated plus maze (EPM), and open field (OF) test occurred at 6, 12 and 18 months of age. Acoustic startle (AS) reflex was also assessed at 6 and 12 months of age. All behavioural testing was conducted during the dark portion of the light:dark cycle, approximately 1 h after lights-off, two hours after the provision of food, and following a 30-min acclimation period to the testing conditions. Testing did not extend beyond the first half of the dark cycle. Mazes/boxes were cleaned with 70% ethanol between tests. A closed-circuit camera mounted above the EPM and OF allowed behaviour to be recorded and tracked with Ethovision XT (Noldus, SDR Clinical Tech, Middle Cove, NSW, Australia) ethological tracking software which was operated in an adjacent room. Locomotor activity, as indexed by the total distance moved (cm) was measured for these tests.

#### 2.3.1. Elevated Plus Maze

Under dim lighting conditions (~100 lux), rats were placed in the center of the maze facing an open arm (50 × 12 cm; l × w; 50 cm wall height for closed arms) and allowed to freely explore the maze for 5 min. The duration and frequency of entries into the open and closed arms of the maze was calculated by Ethovision XT. Entry into each zone was operationally defined as having two paws in the zone. The ratio of open to total arm entries, open to closed arm entries, and open arm duration to total arm duration was calculated as an index of anxiety-like behaviour.

#### 2.3.2. Open Field Test

The OF test was conducted in a large square arena with opaque white Perspex walls (120 × 120 × 50 cm; l × w × h) illuminated centrally from above with a bright light (~400 lux). Rats were placed in the center of the OF under a small compartment that was removed at the commencement of the test. Behaviour was recorded for 10 min. The OF was divided into an outer and centre zone offline using Ethovision, with the size of the central zone being designated as 96 × 96 cm. Entry into the zones was operationally defined as two paws. The duration of time (%) and the frequency of entries into the centre of the OF was calculated. Given all rats commenced testing in the central zone of the OF, the initial latency to leave the centre was removed from the calculation of total time spent in the central zone.

#### 2.3.3. Acoustic Startle

Acoustic startle (AS) was recorded in startle chambers (Med Associates, St. Albans, VT, USA) consisting of an animal holding box. All sessions began with a 5 min acclimation period to background noise (65 dB) followed by 20 presentations of the startle pulse (120 dD, 38 ms, ITI 10–20 s). Startle responses were recorded as startle amplitude and defined as the maximum startle magnitude recording during the 200 ms period following the startle pulse onset. Acoustic startle response (ASR) and short-term habituation were determined by the average startle amplitudes for the first and final 10 startle stimuli presentations, respectively.

### 2.4. RNA Preparation and Sequencing

At 18-months of age, rats were humanely euthanised via carbon dioxide asphyxiation and rapidly decapitated with the aid of a guillotine 2–4 h after lights-out. The hypothalamus, amygdala, pituitary, and adrenal glands were rapidly dissected out with the aid of a rat brain atlas and a 1 mm coronal brain block (Braintree Scientific, Braintree, MA, USA). Tissue was immediately stored in Allprotect^®^ Tissue Reagent (Qiagen, Hilden, Germany) and subsequently placed into a −20 °C freezer. Total RNA was extracted from rat brain tissue and adrenals (*n* = 5/region/group) using E.Z.N.A.^®^ DNA/RNA Isolation Kit (Omega Bio-tek Inc., Norcross, GA, USA), according to the manufacturer’s protocol. Total RNA concentration was determined by using the Nanodrop 2000c spectrophotometer (Thermo Scientific Inc., Waltham, MA, USA), whilst integrity was assessed using Agilent 2200 TapeStation instrument (Agilent Technologies, Santa Clara, CA, USA). RNA sequencing libraries were prepared using TruSeq Stranded mRNA Library Prep Kit (Illumina, Inc., San Diego, CA, USA) according to the manufacturer’s protocol. The libraries were quantified and qualified using the High Sensitivity D1000 Screen Tape on an Agilent 2200 TapeStation instrument. The libraries were normalised, pooled, and subjected to cluster and paired-end sequencing was performed for 150 cycles on a HiSeqX10 instrument (Illumina, Inc., San Diego, CA, USA), according to the manufacturer’s instructions.

### 2.5. Data and Statistical Analysis of Body Weight and Behavioural Data

All animals and data points were included in the analysis. Statistical analysis of body weight and behaviour was conducted using Bayesian generalised linear mixed-effects regression models using subject as the random effect and default priors [26]. Unless specified, model diagnostics indicated convergence and efficiency diagnostic criteria were met [27]. Experimental effects were assessed inferentially using the Sequential Effect Existence and Significance Testing framework (SEXIT) [28,29]. To infer that an effect is not practically equivalent to zero, the SEXIT framework requires the reporting of: the median (*E_M_*), and highest density interval (HDI) of the full posterior of the effect size, the probability of direction of the effect (*D_p_*), and proportion of the effect inside a region of practical equivalence (*ROPE_p_*). For linear models, the region is calculated as: *ROPE_p_* = ± 0.05 *x SD* (*y*), where *SD* (*y*) is the standard deviation of the dependent variable. When less than 2.5% of the full posterior of the effect remains within a region of practical equivalence, the effect is considered not practically equivalent to zero [28,30]. The four requisite statistics are reported as follows: *E_M_* (HDI lower bound, HDI upper bound), *D_p_*, *ROPE_p_*.

### 2.6. Transcriptomic Data Analysis

Analysis of the generated sequencing reads was performed using CLC Genomics Workbench package 22 (QIAGEN) [31] and Galaxy Australia https://usegalaxy.org.au/ (accessed on 1 May 2021) [32], including: quality control of sequencing reads, trimming, mapping and finding the differentially expressed genes. Rat reference genome and its annotation (*Rattus norvegicus*.mRatBN7.2) were downloaded from Ensembl genome browser https://www.ensembl.org/index.html (accessed on 5 June 2021) and used for mapping and expression analysis. Mapping was performed based on the following parameters: mismatch cost = 2, insertion cost = 3, deletion cost = 3, minimum length fraction = 0.8, and minimum similarity fraction = 0.8. Generalized Linear Model (GLM) based on Negative Binomial distribution [33] was employed for differential expression analysis. The *p*-values were also corrected with false discovery rate (FDR) for multiple testing. The use of the GLM allows for curves to be fit to expression values without assuming that the error on the values is normally distributed. Fold changes were calculated from the GLM, which corrects for differences in library size between the samples and the effects of confounding factors. The Wald test was applied to calculate the *p*-values and FDR *p*-value for comparison of all group pairs. *p*-value and *p*-value FDR (corrected) were used for selection of genes with significant differential expression in comparison of long-term CR against the control group in each of the studied brain regions.

Enrichment analysis of Gene Ontology terms in the concept of biological process, molecular function, and cellular component was performed using String webtool https://string-db.org/ (accessed on 20 June 2021) [34]. Multivariate analysis, including principal component analysis (PCA) and clustering, were performed using Minitab Statistical Software 19. PCA analysis was performed using correlation matrix.

### 2.7. Signature Discovery by Attribute Weighting (Feature Selection) Models

Integrated dataset of gene expression (based on fragments per kilobase of exon per million mapped fragments, FKPM) values as well as the additional variable (attribute) of tissue was mined by seven attribute weighting models, including Info Gain Ratio, Rule, Chi Squared, Gini Index, Uncertainty, Relief, and Info Gain. The mentioned models can analyse the numerical values of gene expression as well as the categorical variable of brain region/tissue (hypothalamus, amygdala, pituitary, and adrenal glands). At first, low expressed genes with an average of FKPM < 5 were filtered. The analysis was performed using Rapid Miner Studio Software (RapidMiner version 9, Rapid-I GmbH, Stochumer Str. 475, 44,227, Dortmund, Germany), as previously described [35,36]. Weights of each model was normalised to have the value between 0 to 1, when 0 means non-important and 1 means high importance (responding to long-term CR). The top 20 genes that received the higher weights by seven applied feature selection models (sum of the weights of all models) were selected as the transcriptomic responding signature of brain and adrenal glands to long-term CR.

### 2.8. Text Mining and Integrative Network Analysis of Expressed Genes with Behaviour

We employed literature mining by MedScan [37], a Natural Language Processing (NLP) implemented in Pathway Studio webtool (Elsevier) [38], to shed light on the relations between responding genes in the transcriptomic signature of long-term CR and anxiety, depression, ageing, and HPA axis from full texts of published papers, as previously described [39,40,41]. The following steps were performed in literature mining by Pathway Studio tool: (1) reading sentences in published paper; (2) detection of entities (proteins, behavioural terms, microRNAs, lung cancer, etc.) in the selected sentence; (3) finding the described relationships between entities; (4) identifying the relation type; and (5) adding the detected rule to the SQL database. The title of the paper, authors, publishing year, and Medline (PubMed) reference number were also deposited in the database. The Mammalian + ChemEffect + DiseaseFx database of Pathway Studio (Elsevier) is enriched with proteins, small molecules, stress, and behaviour terms, and disease. Gene Ontology (GO), was used for visualization of results as previously described [41,42,43,44,45]. Mammalian + ChemEffect + DiseaseFx database is updated weekly using cloud technology. The database contains genes/proteins information from human, rats, and mice and is enriched with GO information that can be utilised for finding potential extracellular and secreted proteins as biomarker candidates. The statistics of the database generated for the integrative network analysis of the transcriptomic signature of CR is provided in Table 1. In sum, a highly enriched database with more than 1.4 million entities (including proteins, small molecules, cell process and tissues) and 15 million relations were examined for the transcriptomic signature of CR.

## 3. Results

### 3.1. Body Weight

**Figure 1:**
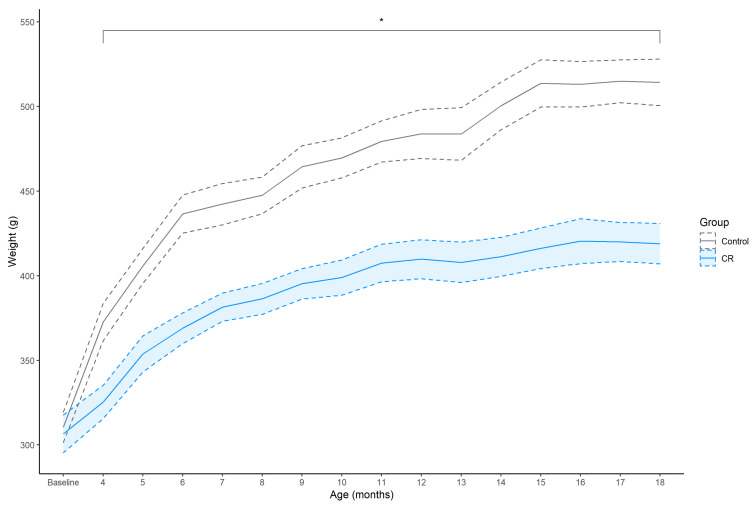
Mean ± SEM (shaded area) weekly body weight (g) of control (*n* = 7) and calorie restriction (CR) (*n* = 8) treated males. Asterisk (*) indicates that between-group effect is not practically equivalent to zero at each time point.

Before the onset of CR at 3-months of age, body weight (g) was similar between the groups; however, thereafter the CR group exhibited an attenuated weight gain compared to *the ad libitum* fed controls from 4- to 18-months of age (Figure 1). The CR group exhibited an approximate 13% attenuated weight gain compared to controls after 1 month of CR, peaking at 19% at 18 months of age, [4 months: *E_M_* = −48.03 (−131.43, 23.68), *D_p_* = 0.99, *ROPE_p_* = 0.02; 18 months: *E_M_* = −95.94 (171.28, 6.86), *D_p_* > 0.99, *ROPE_p_* < 0.01].

### 3.2. Elevated Plus Maze

**Figure 2:**
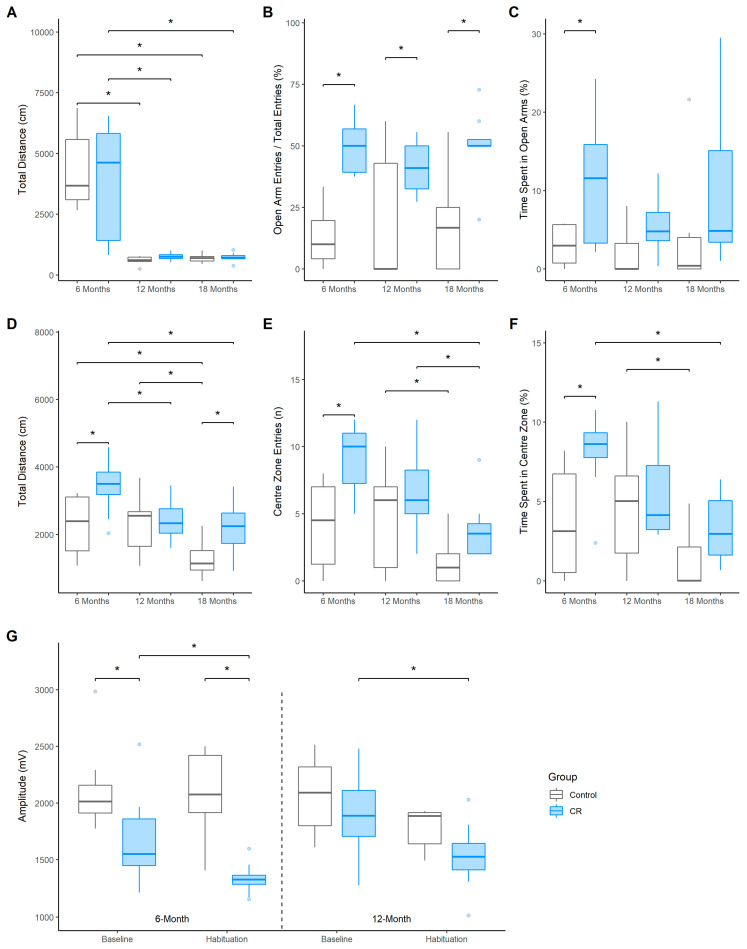
Boxplots indicate median and quartile range across the 6-, 12-, and 18-month of age testing timepoints. (**A**) distance travelled (cm), (**B**) frequency of entries, and (**C**) time spent (%) in the open arms of the EPM; (**D**) distance travelled (cm), (**E**) frequency of entries, and (**F**) duration of time spent (%) in the centre of the OF; (**G**) Mean startle amplitudes (mV) to the startle pulse (120 dB) during baseline and habituation sessions of the acoustic startle test for the control and CR treated males (*n* = 7–8 per group). Asterisk (*) indicates that effect is not practically equivalent to zero.

The total distance travelled (cm) within the EPM was similar between the groups at the 6-, 12- and 18-months of age testing timepoints, with a sharp decline evident for both groups after 6-months of age [Control: 12 months, *E_M_* = −3758.18 (−1076.44, −5876.50), *D_p_* > 0.99, *ROPE_p_* < 0.01; 18 months, *E_M_* = −3680.08 (−1281.02, −6018.82), *D_p_* > 0.99, *ROPE_p_* < 0.01; CR: 12 months, *E_M_* = −3029.76 (−611.21, −5552.26), *D_p_* = 0.99, *ROPE_p_* = 0.02; 18 months, *E_M_* = −3087.39 (−935.01, −5308.28), *D_p_* > 0.99, *ROPE_p_* < 0.01, Figure 2A]. The CR group made more frequent entries into the open arms of the maze compared to controls at all 3 timepoints [6-months old: *E_M_* = −36.61 (−70.7, 3.63), *D_p_* > 0.99, *ROPE_p_* < 0.01; 12-months-old: *E_M_ = −21.49 (−56.32, 14.92)*, *D_p_ = 0.99*, *ROPE_p_ = 0.02*; 18-months old: *E_M_* = −32.83 (−72.24, 3.15), *D_p_* > 0.99, *ROPE_p_* < 0.01; Figure 2B]. Additionally, the CR group spent more time in the open arms of the EPM than controls at 6-months of age [*E_M_* = 7.76 (−6.55, 20.92), *D_p_* = 0.98, *ROPE_p_* = 0.02]; however, only a trend in this direction was demonstrated at 18-months of age (Figure 2C). Notably, the entries into and the time spent in the open arms did not decrease for either group over time.

### 3.3. Open Field

Whilst the distance travelled by the CR animals in the OF was greater compared to controls at 6-months of age, *E_M_* = 1155.58 (−1138.19, 2895.02), *D_p_* = 0.99, *ROPE_p_* = 0.01, and 18-months of age, *E_M_* = 876.02 (−1674.1, 2998.90), *D_p_* = 0.98, *ROPE_p_* = 0.02, this effect was not present at 12-months of age, *E_M_* = 126.92 (−2264.93, 2046.68), *D_p_* = 0.62, *ROPE_p_* = 0.18 (Figure 2D). Furthermore, the control group travelled less at 18 months compared to previous trials (6−18 months, *E_M_* = −938.96 (−2184.39, 559.62), *D_p_* > 0.99, *ROPE_p_* < 0.01; 12−18 months, *E_M_* = −998.19 (−2070.07, 144.34), *D_p_* > 0.99, *ROPE_p_* < 0.01), whilst the distance travelled for the CR group decreased from the 6-month trial (6–12 months, *E_M_* = −975.46 (−2190.33 62.83), *D_p_* > 0.99, *ROPE_p_* < 0.01; 6–18 months, *E_M_* = −1227.11 (−2297.22, −27.87), *D_p_* > 0.99, *ROPE_p_* < 0.01).

Anxiety-like behavioural measures in the OF were found to be partially modulated by CR. The CR group entered the centre of the OF more frequently than control group at 6-months of age, *E_M_* = 5.02 (−1.69, 11.53), *D_p_* > 0.99, *ROPE_p_* < 0.01 (Figure 2E), with both groups entering the centre less frequently at 18-months of age compared to 12-months of age [Control: *E_M_* = −3.13 (−8.74, 1.94), *D_p_* = 0.99, *ROPE_p_* = 0.01; CR: *E_M_* = −2.74 (−7.58, 2.16), *D_p_* = 0.99, *ROPE_p_* = 0.02]. Notably, the CR group also entered the centre less frequently at 18-months compared to 6-months of age, *E_M_* = −5.13 (−11.1, 0.01), *D_p_* > 0.99, *ROPE_p_* < 0.01. Furthermore, the CR group spent a greater time in the centre of the OF compared to controls at 6-months of age, *E_M_* = 4.48 (−2.48, 11.34), *D_p_* > 0.99, *ROPE_p_* < 0.01, although this difference was not evident at 12-months and 18-months of age (Figure 2F).

### 3.4. Acoustic Startle

Baseline AS reactivity, as indicated by the mean startle amplitude (mV) to the first 10 presentations of an acoustic pulse, was reduced in the CR animals at 6-months of age when compared to the free fed controls, *E_M_* = −455.67 (−1346.76, 315.93), *D_p_* = 0.99, *ROPE_p_* = 0.01 (Figure 2G). Furthermore, the reduced startle amplitude in the CR group persisted during the habituation (final 10 startle presentations) session (*E_M_* = −758.96 (−1541.12, 62.77), *D_p_* > 0.99, *ROPE_p_* < 0.01). No group differences were evident for baseline or habituation sessions at 12 months of age. Notably, however, the habituation startle amplitude was lower than baseline for the CR group at both 6 months, (*E_M_* = −343 (−991.25, 269.43), *D_p_* = 0.99, *ROPE_p_* < 0.02), and 12 months of age, (*E_M_* = −371.72 (−1097.56, 527.52), *D_p_* = 0.98, *ROPE_p_* = 0.02), indicating habituation to the startle stimuli in CR animals only.

### 3.5. Tissue-Based Response to Long-Term CR: Genes with Significant Differential Expression in Each Region of Interest

Differential gene expression in each of the regions/tissue examined (hypothalamus, amygdala, pituitary, and adrenal glands) of long-term CR and control groups are presented in File S1. Of the top 10 differentially expressed genes for each tissue (Table 2), genes such as *LOC687780*, *C1qa*, and *Csf1r* were found to be downregulated by long-term CR in all regions examined, with the downregulation significant for all regions, apart from the expression of *C1qa* in the hypothalamus and *Csf1r* in the hypothalamus and amygdala, which demonstrated a trend for this (*p* < 0.09). In contrast, *AC134224.2*, *AABR07044397.1*, and *LOC300308_1* were found to be significantly upregulated under CR conditions in all regions examined. Tissue-dependency was observed for some genes. For example, *Gh1* expression was significantly increased in the hypothalamus by long-term CR; however, its expression was found to be significantly reduced in the amygdala and pituitary.

Multivariate analysis of the top 40 differentially expressed genes (fold changes) responding to CR in each of the areas examined demonstrates a unique tissue-dependent transcriptomic signature of long-term CR (Figure S1). PCA1 and PCA2 efficiently discriminated the amygdala, pituitary, hypothalamus, and adrenal glands, explaining 74.4% of variation in the data collectively. Clustering demonstrated tissue-dependency in response to long-term CR. The amygdala and adrenal glands clustered together at the similarity level of 70.13%, discriminating from the hypothalamus and pituitary.

### 3.6. Tissue-Independent Transcriptomic Signature of Long-Term CR: Application of Attribute Weighting Analysis of Gene Expression

Since the hypothalamus, amygdala, pituitary, and adrenal glands are involved in the regulation of anxiety, development of a tissue-independent transcriptomic signature of long-term CR is desirable. To this end, we employed the 7 attribute weighting models that are capable of analysing both numerical data of gene expression as well as categorical data of tissue type. After filtering low expressed genes, 6245 genes with tissue type (6246 variables in total) were mined for a transcriptomic signature of long-term CR. The weights of each model (Info Gain Ratio, Rule, Chi Squared, Gini Index, Uncertainty, Relief, or Info Gain) were normalised to be ranged between 0 to 1, where 0 means no importance, and a weight equal to 1 demonstrates highest importance of that variable in response to CR. Sum of the weights was used for ranking of genes as well as tissue type.

**Figure 3:**
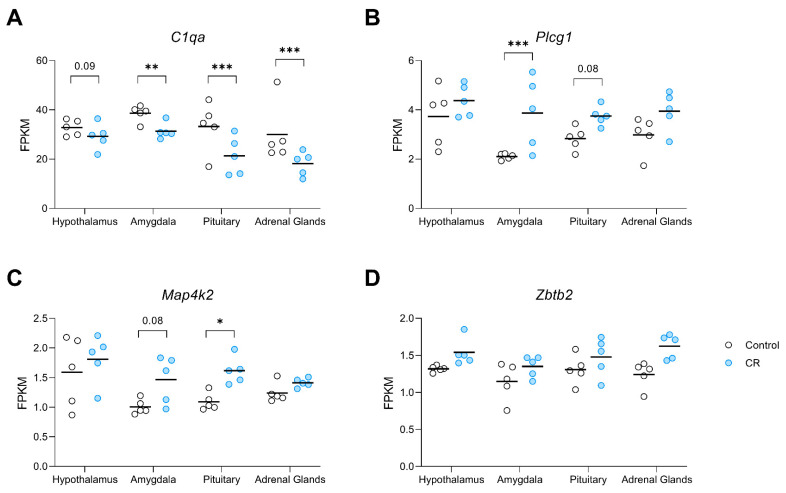
Dot plots visualising the expression of genes in the transcriptomic signature of CR (**A**) *C1qa*, (**B**) *Plcg1*, (**C**) *Map4k2*, and, (**D**) *Zbtb2*, derived from attribute weighting models, in the hypothalamus, amygdala, pituitary, and adrenal glands of control and CR rats (*n* = 5/group/region). Attribute weighting was successful in the selection of genes with a similar trend of response to long-term CR across different tissues. Values are gene expression based on FPKM (fragments per kilobase of exon per million mapped fragments) values. *p* value * ≤ 0.05; ** ≤ 0.01 *** ≤ 0.001.

The rank of genes and tissue type in response to long-term CR using the 7 attribute weighting models is presented in File S2. Out of 6246 mined variables (6245 gene expression data and type of tissue), tissue type received the lowest weight, demonstrating the success of attribute weighting models in developing a tissue-independent signature of CR. The top 20 genes with higher overall weights (sum of weights) are presented as a transcriptomic signature of CR (Table 3). Attribute weighting models highlighted *Zbtb2*, zinc finger and BTB domain containing 2, as the top transcription factor responding to CR, followed closely by *Plcg1*, a gene encoding the protein phospholipase C gamma 1. *Map4k2*, a member of serine/threonine protein kinase family, was also selected by the employed attribute weighting algorithms. Noticeably, *C1qa* (Complement C1q A Chain) was selected by attribute weighting models (Table 3) as well as in the tissue-based differential expression analysis (Table 2), where its expression was significantly decreased in the amygdala, pituitary and adrenals relative to controls, with a similar trend in this direction for the hypothalamus (Figure 3A). *Plcg1*, was found to be upregulated in the amygdala by CR with a strong trend in that direction for the pituitary (*p* = 0.07, Figure 3B). Whereas *Map4k2* demonstrated significant upregulation in the pituitary and a trend in this direction in the amygdala (*p* = 0.08) under CR conditions (Figure 3C). In response to long-term CR, *Zbtb2* showed a pattern of upregulation in all examined tissue (Figure 3D).

Interestingly, Gene Ontology (GO) enrichment analysis of the top 100 genes with higher weights in response to long-term CR showed the significant (*p*-FDR < 0.01) enrichment of “protein serine/threonine/tyrosine kinase activity” molecular function (GO:0004712; File S3). Additionally, in terms of cellular component, GO enrichment analysis demonstrated the signature genes significantly enrich (belong) to specific cellular locations such as the “intracellular organelle”, “intracellular membrane-bounded organelle”, “nucleus”, and “endosome” (File S3). As an example, 64 out of 100 genes belong to GO:0043231: Intracellular membrane-bounded organelle (File S3). This is the first report of the involvement of *Zbtb2* transcription factor and *Map4k2* kinase in response to CR that opens a new avenue for further studies on regulatory mechanisms of brain response to CR.

### 3.7. Text Mining-Based Network Analysis Links Long-Term CR Transcriptomic Signature with Behaviour (Anxiety, Depression, Cognition), Ageing and HPA-Axis

**Figure 4:**
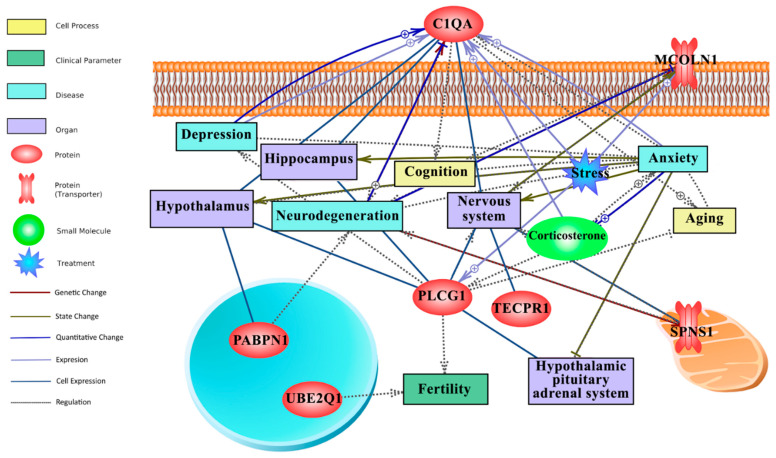
Text mining-derived network of the CR responsive genes, as identified by the attribute weighting analysis, with behaviour (e.g., anxiety, depression, etc.), ageing (e.g., neurodegeneration), regions/systems (e.g., hypothalamus, HPA system), fertility and stress. Detailed relationships, cellular locations, references, and mined sentences are provided in File S4. Integrated network shows that six genes from the attribute weighting analysis (*C1qa*, *Pabpn1*, *Ube2q1*, *Plcg1*, *Tecpr1*, *Mcoln1*) showed relationships with search terms. Among those, *C1qa* and *Plcg1* appeared most important for anxiety and depression. Note: ⨁ denotes positive relation; ⊣ denotes inhibitory relation.

In this study, literature mining was employed to fill the gap between attribute weighting-derived responsive genes to long-term CR, behaviour (anxiety, stress, depression, etc) and the nervous system. Figure 4 illustrates the literature mining-derived network. The cellular locations of expressed genes were retrieved from Gene Ontology. Detailed relationships, cellular locations, references, and mined sentences are deposited in File S4. In literature mining, the number of mined sentences in references that support a particular relationship is an index of confidence level where 3, 2, and 1 stand for high, medium, and low confidence, respectively [46,47]. For example, in the constructed literature mining network (Figure 4), the positive relationship between *C1qa* and neurodegeneration (C1QA -- + > neurodegeneration) and ageing (C1QA -- + > ageing) is robust, supported by 22 and 19 mined sentences in published papers, respectively (File S4).

Within the top 20 genes of the CR transcriptomic signature, *C1qa* and *Plcg1* were identified as hubs in the network that link long-term CR transcriptomic signature to ageing, the HPA axis, the nervous system, stress, behaviour, neurodegeneration, and fertility (Figure 4). *Plcg1* was found to be positively linked to stress (4 mined sentences) and as having a regulatory role in fertility and depression, although the latter yielding medium to low confidence scores, 2 and 1, respectively. Interestingly, *C1qa* has largely a positive relationship (regulation, expression, quantitative change) with anxiety, stress and depression, collectively yielding a high confidence score (10 mined sentences; File S4). The downregulated expression of *C1qa* by long-term CR (Figure 3) and its extracellular location as a secretory protein flag *C1qa* as a potential biomarker of CR mediated changes to anxiety and depression.

## 4. Discussion

Here, we report on the effects of mild CR on the life-time trajectory of anxiety-like behaviour in male Hooded Wistar rats as well as transcriptome-wide gene expression within brain regions (hypothalamus, amygdala, pituitary) and tissue (adrenal glands) implicated in the regulation of anxiety in aged rats. Attribute weighing algorithms were employed to select a subset of genes as a tissue-independent transcriptomic signature of long-term CR. Then, we utilised literature mining to link the responsive genes in long-term transcriptomic signature of CR with depression/anxiety and nervous system.

The results from the present study show that a long-term adult-onset CR of 25% results in an anxiolytic behavioural profile in male rats across early, middle, and late adulthood, as evidenced by greater entries into the open arms of the EPM at 6-, 12-, and 18-months of age. CR modulation of anxiety-like behaviour appears to be the strongest during early adulthood, given at 6-months of age the CR group additionally spent more time on open arms of the EPM, made more entries into, and spent more time in the centre of the OF than *ad libitum* fed controls. The AS response was attenuated in CR animals at both 6- and 12-months of age, further indicating an anxiolytic response of long-term CR.

The robust anxiolytic effect observed in restricted rats at 6-months of age is consistent with the bulk of the research to date examining adult-onset CR in rats [11,12,13,14] and extends on these to find that this effect is sustained after four months of CR. The general pattern of our findings is congruent with [19] where an anxiolytic effect was evident in late adulthood (18–20-months) and extends on these to report the same effect with a milder level of CR. Our findings are inconsistent with [18]; however, where an anxiogenic profile was identified at ~6 and 20 months. Methodological differences in degree, duration and in the age that CR was initiated may account for these inconsistent observations. For instance, it is possible that the juvenile onset of CR in [18], versus adult onset in the current study, accounts for the conflicting results at the later time points, particularly as early life CR can program anxiogenic profiles in adulthood [48]. Furthermore, the differences in the behavioural profile within our study and previous studies may highlight a sex effect.

It is unlikely that the moderated effect observed in middle and late adulthood is a consequence of the reduced anxiolytic effects typical of re-testing [49], as re-testing was conducted in novel rooms and more than 28 days apart; conditions which have been reported to ameliorate this effect [50]. It is more likely that the moderated effect at the later time-points is due to the sharp decline in locomotion observed after 6-months of age potentially masking stronger treatment effects. The EPM and OF tests are ethologically based tests of forced exploration and are thereby locomotion-dependent and sensitive to changes in locomotion [51]. Consequently, these tests may not have been sensitive enough to robustly assess anxiety-like behaviour in an ageing population. To address the issue, we included the acoustic startle test as a non-locomotive indicator of anxiety-like behaviour. Though not a direct test of anxiety, increased anxiety states can augment the acoustic startle response and interfere with the habituation of startle [52,53]. As such, the reduced acoustic startle response observed at 6-months and the habituation of the startle response at 6- and 12-months in the CR group complements and substantiates the results from the locomotion-dependent test results, indicating a protracted anxiolytic effect of CR well into adulthood. These phenotypic reductions in anxiety across the life-time trajectory have clinical implications for the capacity of CR to buffer against not only anxiety, but a host of other psychiatric disorders where CR-mediated effects have been noted, such as depression and drug addiction [54,55].

To elucidate the mechanisms of CR-induced behavioural plasticity, we measured transcriptome-wide gene expression in brain regions and tissue involved in the regulation of anxiety at 18-months of age, 6 to 12 months following the strongest behavioural effects observed in anxiety-like behaviour. Among the top 40 differentially expressed genes, several long intergenic non-coding RNAs (lincRNAs) were altered by CR indicating epigenetic modifications. LincRNAs have been associated with nuclear stability and thus may play an integral role in neural health [56]. Furthermore, some lincRNAs have been identified as biomarkers or therapeutic targets [56]. Also, within the top 40 differentially expressed genes, *Csf1r*, here observed to be downregulated in the pituitary and adrenal glands in response to long-term CR, has documented associations with anxiety-related behaviour, principally through its role in immunomodulation [57,58,59,60]. For instance, administration of a *Csf1r* antagonist has been found to abrogate stress-induced anxiety-like behaviour in rodent studies through the depletion of microglia and concomitant reduction in neuroinflammation [58,59].

Additionally, we observed downregulation of *C1qa*, a gene also involved in inflammation-related function, in the amygdala, pituitary, and adrenal glands. Attribute weighting models also identified *C1qa* as a tissue-independent transcriptomic signature of long-term CR and literature mining-based network analysis identified it as a hub in the constructed network, linking this long-term CR responsive gene not only to anxiety, but also depression, stress, ageing, and neurodegeneration. In terms of documented links with anxiety, literature mining revealed a robust positive relationship between *C1qa* and anxiety, where increased *C1qa* expression is associated with more anxious phenotypes [57,61,62,63]. Collectively, our data and existing research literature suggests that CR may regulate anxiety-like behaviour through an attendant reduction in inflammatory signalling pathways [64,65]. This finding is congruous with the putative role of systemic inflammation in the modulation of affective behaviour, as well as the aetiology and maintenance of anxiety disorders (see [66] for a review). Notably, the significant downregulation of *C1qa* in our tissue-based expression analysis, the identification of this gene as a tissue-independent transcriptomic signature, and as a hub in our literature-mining based network, coupled with its extracellular location, candidate *C1qa* as a biomarker of an efficient long-term CR diet linked with reduced anxiety-like behaviour in aged animals.

A further candidate identified as a tissue-independent transcriptomic signature of long-term CR is upregulation of *Plcg1*, a gene involved in brain development and synaptic transmission. Literature mining-based network analysis identified *Plcg1* as a hub in the constructed network, linking this gene to anxiety, depression, stress, the nervous system, and fertility, with 2 mined sentences identified linking this gene to anxiety-a medium confidence result. One such study found that increased *Plcg* pathway activation in a transgenic mouse strain overexpressing the full-length neurotrophin receptor trkB, led to a diminution of anxiety-related behaviour [67]. Additionally, *Map4k2*, also upregulated here and identified as a transcriptomic signature, has some links to anxiety associated with early life stress [68].

Shortlisting of genes, such as *C1qa*, that establish the crosstalk between CR, anxiety, depression, and neurodegeneration is of high clinical importance in the following contexts: (1) biomarker discovery for anxiety as well as other disorders; (2) discovery of genomic variants in response to CR, especially those with risk of anxiety, depression and neurodegeneration; (3) drug discovery/repurposing that can simulate/mimic the beneficial effects of long-term CR by activation of the transcriptomic signature.

## 5. Conclusions

This is a pioneering study that reveals phenotypic reductions in anxiety-like behaviour across the life-time trajectory of rats and unravels the transcriptomic signature of both coding and non-coding RNAs in the key regulatory tissues of anxiety in response to long-term CR. Comprehensive feature selection by attribute weighting algorithms was successful in developing a transcriptomic responsive signature to long-term CR across the hypothalamus, amygdala, pituitary, and adrenal glands. Crosstalk between genes of this transcriptomic signature with anxiety, depression, ageing, and neurodegeneration was established by literature mining. Collectively, we identify *C1qa* as a potential candidate biomarker of long-term CR linked to phenotypic reductions in anxiety. Understanding the molecular mechanisms and benefits of this dietary manipulation highlights therapeutic targets not only for anxiety, depression, and neurodegeneration, but will also contribute significantly towards unravelling the global anti-ageing capacity of CR.

## Figures and Tables

**Table 1:** Statistics (number of mined texts) of Mammalian + ChemEffect + DiseaseFx database (June 2022) used for integrative network analysis of the CR transcriptomic signature with stress and behaviour (e.g., anxiety, depression) in this study. The database is constructed by text mining of full text of biomedical papers using Natural Language Processing (NLP).

Category	Sub-Category	Number
Entities	Proteins	144,002
	Cell Objects, such as extracellular organelle	632
	Cell process, including ageing	14,153
	Cells, including cell lines information	4297
	Clinical parameters, including energy consumption, energy expenditure, energy intake, and fertility	5452
	Complexes, such as fatty acid beta-oxidation multienzyme	994
	Diseases, including depression and anxiety	23,016
	Pathogens	607
	Small molecules, including drugs	1,057,758
	Treatments, including stress	88
	Tissue, such as hypothalamus	3908
	Functional Classes, such as Fe transporter	5570
	Genetic Variants	168,360
	Virus	25,323
	Tissues, such as lung mucosa	588
	Total Entities	1,454,748
Relations	Binding relations	1,176,779
	Biomarker relations	155,684
	Cell expression relations	1,544,338
	Chemical reaction relations	64,482
	Clinical trial relations	133,031
	Direct regulation relations	798,934
	Expression relations	1,061,638
	Functional associations	2,120,740
	Genetic change relations	481,568
	Molsynthesis	185,956
	Moltransport	295,541
	Promoter binding	52,413
	Protein modification relations	85,659
	Quantitative change relations	533,337
	Regulation relations	6,644,356
	State change relations	174,659
	MicroRNA effects	71,649
	Total Relations	15,580,764

**Table 2:** Top 40 differentially expressed genes responding to long-term CR in the hypothalamus, amygdala, pituitary, and adrenal glands (tissue-based signature of long-term CR). Top 10 genes for each tissue are presented. Negative values (−) of fold change show downregulation by long-term CR treatment, and positive values represent upregulation.

Gene Name	Ensembl Identifier	Fold Change			
		Hypothalamus	Amygdala	Pituitary	Adrenal Glands
*LOC687780*	20982	−1.83 ***	−1.81 **	−1.83 ***	−2.1 ***
*AC134224.2*	62155	1.44 **	3.66 ***	2.88 ***	1.46 **
*AABR07044397.1*	50998	3.31 *	7.47 **	5.97 ***	285.24 ***
*LOC300308_1*	48230	1.72 ***	4.44 ***	1.8 **	1.64 *
*C1qa*	12807	−1.21	−1.37 **	−1.75 ***	−1.87 ***
*Csf1r*	18414	−1.24	−1.23	−1.35 *	−1.84 ***
*Gh1*	11207	3.29 ***	−2.03 **	−1.51 ***	−1.24
*Snhg11*	36802	1.39	3.73 ***	2.06 ***	1.31
*Mt-co3*	30700	−1.33	−1.25	−1.87 ***	−1.5 ***
*Cacng8*	57848	1.36 *	2.17 ***	−1.48	3.86 *
*Unc13a*	18452	1.26	1.45 ***	2.26 ***	1.28
*AABR07056156.1*	61013	1.47 *	3.26 ***	1.63	1.37
*Abcc5*	29178	1.26	2.45 ***	1.97 ***	1.14
*Lpin3*	16636	1.31	4.16 ***	1.53 ***	1.13
*Mbp*	16516	−1.37	−1.57 **	−3.17 ***	1.13
*AABR07033720.1*	61472	2.56 ***	2.61 *	1.42	−1.72
*Nr4a2*	05600	1.19	−1.22	−1.53 *	3.23 ***
*Cfd*	33564	−1.34	−2.31	−2.93	−2.52 ***
*Sppl2a*	11652	−2.01 ***	−1.08	−1.5 **	−1.45
*Mcc_1*	62232	−92.32 ***	1.15	NE	NE
*Myorg*	23208	−1.52 ***	−1.5 *	−1.43	1.06
*Plk5*	34102	1.57	1.95 **	2.14 ***	1.12
*Rpp30*	18718	1.11	3.14 **	1.64 **	1.14
*Bpifa1*	13859	1.98	3.86	62.13 ***	−2.87
*L3mbtl1*	07044	1.52 *	5.24 ***	2.61 ***	−1.02
*AABR07065531.26*	59660	1.22	4.38 ***	1.63 *	−1.11
*Scx*	21812	8.03 **	7.59	−1.09	11.05 ***
*Ints10*	55331	1.02	2.58 ***	1.49 ***	1.2
*Mobp*	18700	−1.3	−1.45 *	−16.55 ***	1.01
*Tspoap1*	07957	1.04	1.15	1.96 ***	1.42
*Tmem125_2*	45872	−6.98 ***	1.75	1.14	NE
*Slc17a7*	20650	3.89 ***	1.4	1.07	−4.61
*AABR07000411.1*	55789	3.63 ***	2.31	1.17	1.07
*Nadsyn1*	20736	1.14	1.23	1.95 ***	1.1
*Adamts18*	11575	2.79 ***	−1.03	3.35	1.34
*Acsm5*	31211	1.08	1.02	1.25	−7.91 ***
*Ddn*	59605	1.13	1.12	−16.74 ***	1.02
*Cwc25*	04091	1.04	1.02	1.09	3.17 ***
*Cd300c2*	46216	1.06	−1.02	−1.13	−2.7 ***
*LOC690507*	15637	NE	NE	NE	9.5 ***

Note: Ensembl identifier Prefix: ENSRNOG000000; NE: Not Expressed; *p* value * ≤ 0.05; ** ≤ 0.01 *** ≤ 0.001.

**Table 3:** Transcriptomic signature of long-term CR derived from mining of expression of 6246 genes and tissue type by attribute weighting (feature selection) models. Weight of each variable in each model varies from 0 to 1, where 1 shows highest response to long-term CR. Top 20 genes with higher overall weights (sum of weights) are presented. Tissue type received the lowest weight, demonstrating the development of tissue-independent signature by attribute weighting models.

Rank	Attribute	Gene Name	Weight							Sum of Weights
			Info Gain Ratio	Rule	Chi Squared	Gini Index	Uncertainty	Relief	Info Gain	
1	ENSRNOG00000019544	*Zbtb2*	1.0	0.8	0.9	1.0	0.9	0.6	1.0	6.1
2	ENSRNOG00000062155	*AC134224.2*	0.8	0.5	0.9	0.9	0.8	0.9	0.8	5.6
3	ENSRNOG00000051490	*Plcg1*	0.6	1.0	0.8	0.6	0.7	0.9	0.6	5.2
4	ENSRNOG00000002194	*Coq2*	0.9	0.6	0.7	0.9	0.7	0.6	0.9	5.1
5	ENSRNOG00000009990	*Zranb2*	0.8	0.3	0.8	0.8	0.8	1.0	0.8	5.1
6	ENSRNOG00000000975	*Mcoln1*	0.6	0.6	1.0	0.6	0.9	0.8	0.6	5.1
7	ENSRNOG00000021061	*Map4k2*	0.6	1.0	0.8	0.6	0.7	0.8	0.6	5.0
8	ENSRNOG00000042258	*RGD1561157*	0.9	0.6	0.9	0.7	0.9	0.4	0.7	5.0
9	ENSRNOG00000030721	*Fbrsl1*	0.7	0.4	0.7	0.8	0.7	0.9	0.7	4.9
10	ENSRNOG00000053405	*Rpap3*	0.7	0.2	0.9	0.7	0.9	0.8	0.7	4.9
11	ENSRNOG00000001010	*Tecpr1*	0.7	1.0	0.7	0.5	0.6	0.7	0.6	4.8
12	ENSRNOG00000057284	*Cenpb*	0.8	1.0	0.8	0.6	0.7	0.3	0.6	4.8
13	ENSRNOG00000052539	*Prpf40b*	0.6	1.0	0.8	0.6	0.7	0.5	0.6	4.7
14	ENSRNOG00000017621	*Spns1*	0.7	0.4	0.7	0.8	0.6	1.0	0.7	4.7
15	ENSRNOG00000025711	*Spout1*	0.6	1.0	0.7	0.6	0.6	0.7	0.5	4.7
16	ENSRNOG00000020791	*Ube2q1*	0.8	1.0	0.8	0.6	0.8	0.1	0.6	4.7
17	ENSRNOG00000010732	*RGD1561590*	0.7	1.0	0.7	0.5	0.7	0.5	0.6	4.7
18	ENSRNOG00000042195	*Pabpn1*	0.7	0.4	0.8	0.6	0.7	1.0	0.5	4.6
19	ENSRNOG00000014044	*Pank4*	0.7	0.5	0.7	0.7	0.7	0.7	0.7	4.6
20	ENSRNOG00000012807	*C1qa*	0.6	0.9	0.8	0.6	0.7	0.5	0.5	4.6
6246	Region/Tissue		0.0	0.0	0.0	0.0	0.0	0.7	0.0	0.7

## Data Availability

Data is contained within the article or supplementary material. The data presented in this study are available in [insert article or supplementary material here].

## Supplementary Materials

The following supporting information can be downloaded at: https://www.mdpi.com/article/10.3390/nu14214670/s1, File S1: Differentially expressed genes; Figure S1: Multivariate of tissue-dependent signature; File S2: Attribute weighting data; File S3: Gene ontology enrichment analysis; File S4: Attribute weighting genes references literature mining.
