# Supplementary figures and images for: Long-Term Calorie Restriction Alters Anxiety-like Behaviour and the Brain and Adrenal Gland Transcriptomes of the Ageing Male Rat
Source: Nutrients. 2022 Nov 4;14(21):4670. doi: 10.3390/nu14214670 (PMC9654051; doi:10.3390/nu14214670)

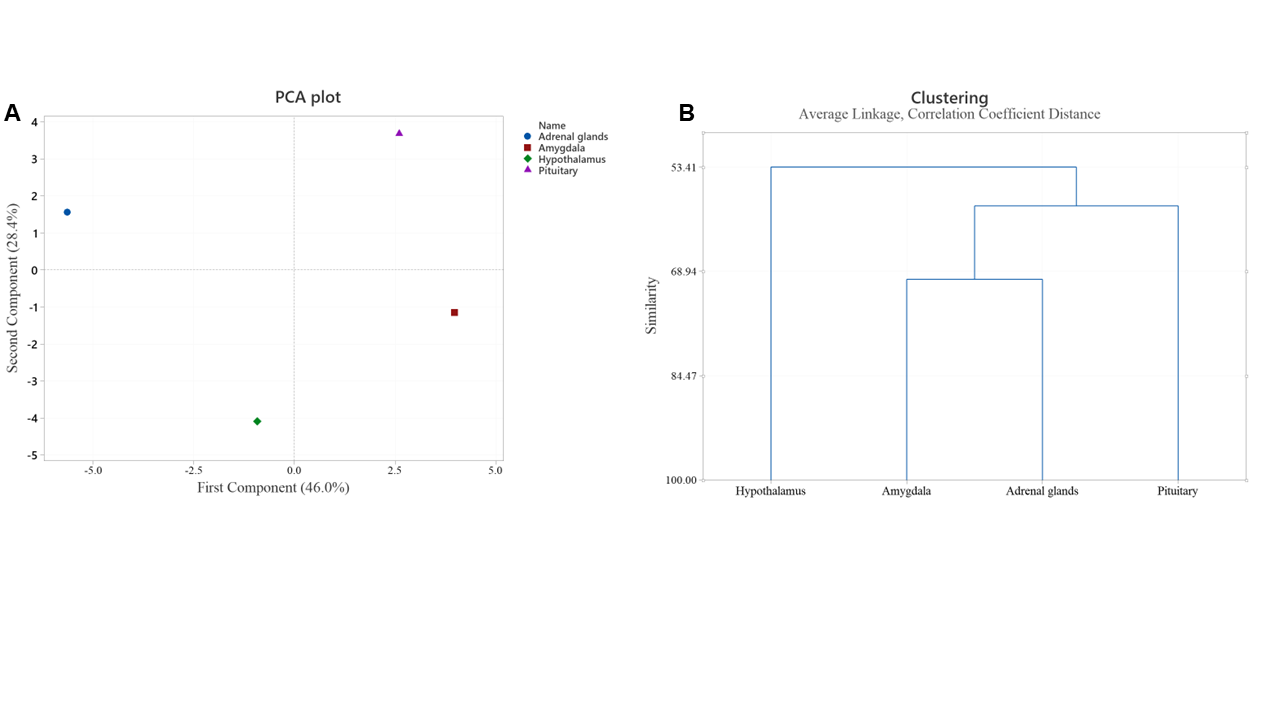

Supplement: Supplementary file 1 [file nutrients-14-04670-s001.zip › Final Supplementary/Figure S1 Multivariate of tissue-dependent signature.tif]
